# A potential difference for single-particle cryo-EM

**DOI:** 10.1107/S2052252519014556

**Published:** 2019-11-01

**Authors:** Peter B. Rosenthal

**Affiliations:** a Structural Biology of Cells and Viruses Laboratory, The Francis Crick Institute, 1 Midland Road, London, NW1 1AT, UK

**Keywords:** single-particle cryoEM, electron cryomicroscopy, low-dose electron microscopy, direct detectors

## Abstract

Nydenova *et al.* [(2019), *IUCrJ*, **6**, 1086–1098] determine structures of frozen-hydrated protein and nucleic acid assemblies using 100 keV electrons, and describe characteristics of electron microscopes designed to exploit advantages of a lower operating voltage for single-particle cryo-EM.

Advances in experimental and computational methods for single-particle cryogenic electron microscopy (cryo-EM) have transformed the field of structural biology by making it possible to calculate high-resolution maps of proteins and nucleic acid assemblies without crystals. Efficient electron detectors and movie-mode data collection have been key to the resolution jumps that have been achieved for low molecular mass, asymmetric specimens. However, cryo-EM has not yet reached its theoretical limit for structure determination from small numbers of particles or from small molecular mass specimens (Henderson, 1995 ▸). Better imaging and computation together are helping to identify and hopefully find solutions to problems, such as beam-induced movement, that may further enhance this already successful technique.

Cryo-EM structure determination proceeds by recording and averaging low dose images of frozen-hydrated specimens embedded in a thin layer of vitreous ice. The structural information in images is related to the ratio of useful elastic electron scattering events to inelastic events that only cause damage and do not contribute to image contrast. A recent study (Peet *et al.*, 2019 ▸) reports new measurements of these electron–specimen interactions for 100 keV and 300 keV electrons, the energy range of electrons commonly used in biological microscopy. The study concludes that for a thin specimen of thickness of about 300 Å, *e.g.* protein molecules in a somewhat larger ice layer, the optimal choice of voltage is 100 kV. For thicker specimens, including many studied in the growing field of cryotomography, higher voltage is beneficial.

This conclusion contrasts recent practice in single-particle cryo-EM, where higher electron acceleration voltages have been favoured because of their greater penetration, reduced multiple scattering and less sensitivity to electron optical aberrations and specimen charging. Indeed, most high-resolution single-particle structures come from 300 keV instruments equipped with a coherent field emission gun (FEG) source and with direct electron detectors (and often an energy filter). There have been studies emphasizing that high-resolution structures can be obtained at 200 keV (Herzik *et al.*, 2017 ▸, 2019 ▸). Furthermore, the efficient direct electron detectors in current use in cryo-EM have been optimized for 300 keV and perform well at 200 keV. At lower energies, backscattering of electrons leads to degradation of signal.

In the article by Naydenova *et al.* in this issue of **IUCrJ** (Naydenova *et al.*, 2019 ▸), the same research team has followed their earlier analysis with a practical demonstration that structure determination of challenging biological specimens can be performed using 100 keV electrons. Their experiments combine all the necessary instrument features and show that many of the problems associated with low voltage can be addressed. The study was performed on a 200 keV microscope with a coherent FEG electron source, though operated at 100 keV. Commercial instruments with lower voltage (a maximum 100 or 120 kV) are not equipped with FEG sources. A key feature of the new study is the use of a hybrid-pixel detector (Faruqi & McMullan, 2018 ▸), developed for X-ray diffraction studies, the Dectris EIGER (Dinapoli *et al.*, 2011 ▸). Imaging and reconstruction are applied to five single-particle specimens of varying sizes and masses: hepatitis B virus capsid, bacterial 70S ribosome, DPS (DNA protection during starvation protein), catalase and haemoglobin (see Fig. 1 ▸). Notably, the DPS protein (220 kDa), was resolved to 3.4 Å.

Additional concerns have caused pessimism about high-resolution work at low voltage. There is a greater effect of beam tilt on image phases at lower voltage. However, beam tilt, as well as other aberrations, is modelled successfully by the software used for reconstruction in this study, *RELION-3* (Zivanov *et al.*, 2018 ▸). Another potential problem at low voltage is that the depth of field (or equivalently, the greater curvature of the Ewald sphere) becomes important for thick specimens at high resolution, and the images are no longer true projections of the structure. Though not a concern at the resolutions reported in the present study, computational correction has been demonstrated (Russo & Henderson, 2018 ▸; Wolf *et al.*, 2006 ▸). The effect of chromatic aberration is worse at lower voltage and ultimately limits resolution.

This is just the beginning for high-resolution work at 100 keV: though the performance of the detector was excellent, its area was small (1030 × 514 pixels), so an inefficient way of obtaining the large number of particles ideally required for high-resolution work. Nor did the detector count individual electrons. The new results reported here should fuel research on the detectors optimized for lower voltage and microscope platforms that possess features proven to be important in their higher voltage relatives.

The authors describe requirements for a future ‘ultimate’ single-particle microscope operating at 100 kV for structure determination to better than 2 Å. With coherent illumination and a large pixel format electron counting detector, the authors specify several ways in which the required small chromatic aberration may be achieved. But perhaps of greater immediate impact, the authors propose a microscope optimized for single-particle data collection to 3 Å or somewhat better, still capable of obtaining near-atomic maps and models, but also suitable for specimen screening. In many investigations, as in the one reported here, sample preparation is not uniformly ideal. Until sample preparation problems are solved more generally, optimization requires significant time on a lower-end screening instrument, but can ensure subsequent success on a higher-end instrument. To be predictive for success at high resolution, the standard for a screening instrument is still quite high. More than showing the shape of particles in ice, it ideally allows one to detect high-resolution features (secondary structure) in 2D image averages, an early step in image analysis, and identify a wide range of orientations, a requirement for isotropic resolution. Voltage is a surrogate for size and cost, both for the instrument and environment to house it. The authors envision that such affordable instruments (and available software) will make cryo-EM more widely accessible. This will reduce some of the (high) tension associated with the rapid growth of the field.

## Figures and Tables

**Figure 1:**
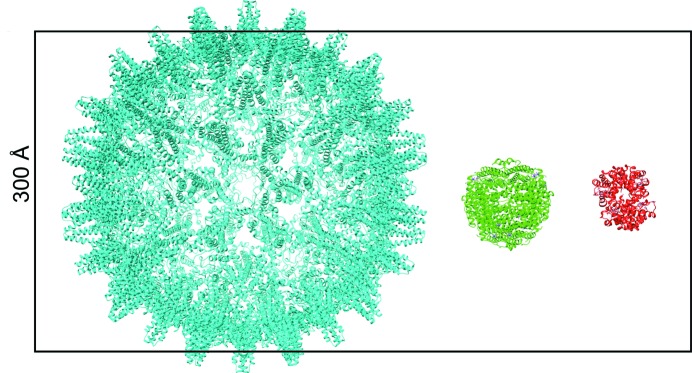
Three of the five single-particle specimens studied using 100 keV electrons in Naydenova *et al.* (2019 ▸) showing relative sizes: Hepatitis B virus capsid (cyan), DPS (green) and haemoglobin (red). The vertical dimension of the bounding box is 300 Å.
